# A Potential-Induced Transformation in the Double Electrical Layer on the Rhenium Electrode in Alkali Chloride Melts

**DOI:** 10.3390/ma14206009

**Published:** 2021-10-12

**Authors:** Ekaterina V. Kirillova, Victor P. Stepanov

**Affiliations:** Institute of High Temperature Electrochemistry, Russian Academy of Sciences, Akademicheskaya Str. 20, 620137 Yekaterinburg, Russia; v.stepanov@ihte.uran.ru

**Keywords:** rhenium, alkali chlorides, potential, electrostatic adsorption, chemisorption

## Abstract

Structural transformations in the adsorption layer caused by an electric potential are investigated using the experimental data on the capacitance of a double electric layer for a rhenium electrode in molten sodium, potassium and cesium chlorides at 1093 K. Likening the double electric layer to a flat capacitor, as well as the effective length of the shielding of the electrode charge and changes in the charge sign depending on the applied potential are estimated. It is found that near the minimum potential of the capacitance curve, the shielding length decreases proportionally to the square of the potential due to the deformation of the double layer. The deformation reaches critical values at the potentials of −0.65, −0.38 and −0.40 V for the Re|NaCl, Re|KCl and Re|CsCl systems respectively, and decreases sharply at more positive potentials. The analysis of the dependence of the charge density on the electrode revealed the effect of shielding of potential-induced rhenium cations by salt phase anions. The strong Raman-active Re–Cl stretching mode was observed at 292 cm^−1^. This can be explained by the transfer of anions across the interface resulting in the formation of ordered layers of ion associations (possibly, ReX_n_^(n − 1)−^) on a positively charged surface.

## 1. Introduction

Recently, two metals, namely rhenium and iridium, have attracted the attention of researchers because of their two technologically important properties. Rhenium, being the most refractory metal after tungsten, retains good plastic properties at a fairly high temperature. The intensive oxidation at temperatures above 850 K is the key disadvantage that limits the use of rhenium products in oxidizing environments. Iridium, on the contrary, can be considered as a protective element against both thermal and chemical attacks, due to its excellent chemical stability and resistance to oxidation [1,2,3,4,5]. Thus, it is reported that rhenium samples with approximately 100 µm thick iridium coatings withstand tests in an air atmosphere at 2273 K for 3 h and maintain good adhesive properties of the coatings [2]. The combination of these properties makes the Ir-Re composite material very promising for use in products and devices operating under extremely harsh conditions at high temperatures (up to 2400 K) and chemically active atmospheres, in particular, for manufacturing low-thrust engines using high-energy fuel [6,7,8,9].

Taking into account the problems of metallurgical processing when working with refractory metals, the electrochemical deposition of compact iridium and rhenium layers, as well as alloys based on them from molten electrolytes, is widely promoted in the scientific literature. The results of these studies have shown that variation of the electrolyte composition [8,9,10,11], temperature [9,10,11,12,13,14] and current density [8,10,11,12,13] makes it possible to obtain metallic coatings with a controlled structure, chemical and phase composition [10,12,13,14,15,16,17], including seamless multilayer products (Ir-Re-Ir) of a desired shape [6,9]. As a rule, the electrolytic formation of products composed of refractory metals is carried out in refining baths, when a soluble anode is the source of the working metal.

It is known that during the anodic dissolution of metals at certain temperatures and current densities, the passivation of anodes takes place. It significantly complicates the production of cathode deposits of the required structure [17]. The nature of the formation of the passivation layer on the anode has not been precisely established yet.

This phenomenon might be explained by the behavior of salt phase ions at the electrode surface in the region of anode potentials. The unusual state of anions was first mentioned in [18,19,20,21] focused on the capacitance of a double electric layer on solid Pt and Au electrodes in molten alkali chlorides. This “unusual state” was recorded as a maximum on the anode branch of the capacitance curve. The authors explained such behavior by the adsorption of the chlorine anion. In the follow-up studies on the gold electrode in molten alkali chlorides, bromides and iodides [22,23,24,25,26], the anomalous course of the capacitance curve could not be explained within the framework of electrostatic adsorption. Therefore, it was assumed to take into account the chemical interaction between the anions and the charge carriers on the electrode. However, this assumption was not supported by the results of other research methods. Nevertheless, the need to consider the interaction between the salt phase ions and the electrode to explain the abnormal change in the capacitance depending on the potential is indicated in a number of theoretical papers [27,28,29]. With regard to the rhenium electrode, it is of interest to obtain the missing information about the possible stages of anions’ adsorption at a charged metal surface. The present paper is focused on the determination of transformations that occur at the interface of a solid rhenium electrode and molten sodium, potassium and cesium chlorides based on the analysis of the dependence of the double electric layer capacitance on the applied electric potential, which was obtained by the impedance method. The behavior of the halide anion at the charged electrode/electrolyte interface is described using the potential dependences of the charge density of the electrode and the deformation of the double electric layer. A two-stage adsorption model, which includes the anion transfer from the salt phase to the electrode, is discussed. The results of spectroscopic studies of the electrode surface are used to verify the model.

## 2. Materials and Methods

### 2.1. Preparation of Materials

Commercial powdery NaCl, KCl and CsCl (purity 99.5–99.8%) were preliminary kept under vacuum for 5 h, first at room temperature, and then they were heated and melted in pure helium to remove the adsorbed water. The fused salts were subjected to six-fold zone melting in pure argon to remove chemical impurities [30] at the facility described in [31]. According to [30], the concentration of residual impurities in the sample after such purification is “less than ten particles per billion”.

The working electrode face, that was supposed to be in contact with the melt, was treated as follows: it was polished to a mirror shine by a silicon carbide sanding paper, washed with distilled water and acetone (purity 99.5%) and dried in air. This procedure has proven itself well in electrochemical experiments with compact rhenium [2,3].

### 2.2. Cyclic Voltammetry

The electrochemical investigations were carried out using a potentiostat-galvanostat Parstat 2273 (Princeton Applied Research, Oak Ridge, TN, USA) at the frequency of an alternating signal of 10 kHz. Before each experiment, the dependence of the polarization current on the applied potential was analyzed to determine the electrochemical cleaning of the electrode surface and the potential interval free of any discharge-ionization processes. All experiments were performed in an argon atmosphere.

### 2.3. Differential Capacitance Determination

The three-electrode experimental unit of the following design was used:

(i). A rhenium bar (99.99% purity) served as a working electrode with a surface area of 0.5 cm^2^,
(ii). A glassy-carbon plate located at the bottom of the crucible with the sample melts was used as a counter electrode,
(iii). A lead pool under the NaCl + KCl + 3 wt.% PbCl_2_ electrolyte in a quartz capsule served as a reference electrode.

**Figure 1:**
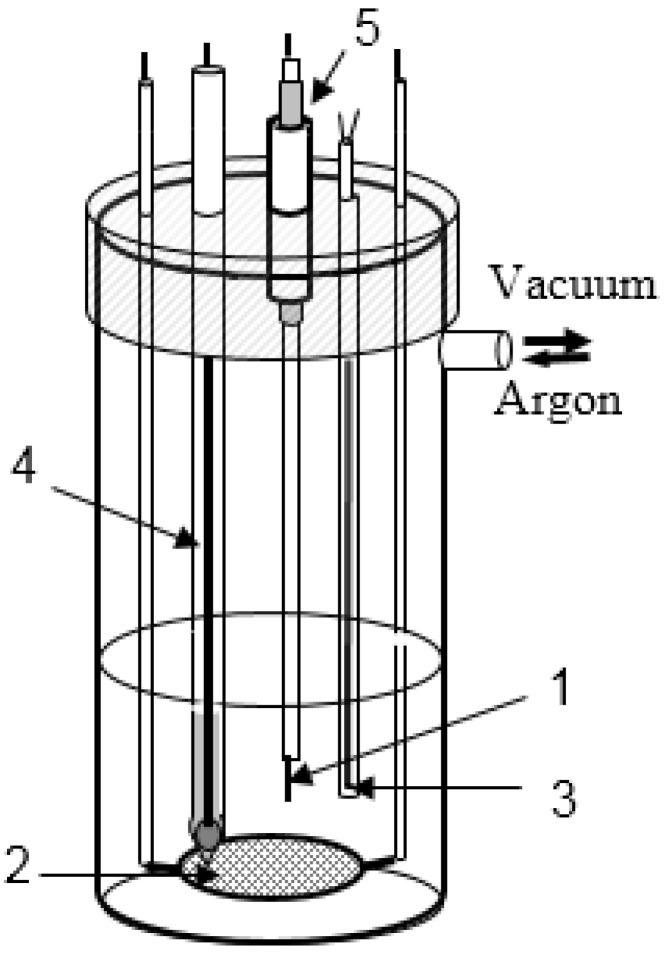
Electrochemical set-up. Working electrode (**1**), counter electrode (**2**), thermocouple (**3**), reference electrode (**4**), glass joint (**5**).

This unit together with the Pt/Pt-Rh thermocouple was located in a quartz tube closed with a heat-resistant rubber stopper, which was used as a working cell (Figure 1). The weighted portion of the studied salt was placed at the bottom of the quartz tube. The heating component of the experimental unit included a tube resistance furnace. The Pt/Pt-Rh thermocouple located in the immediate vicinity of the heating section was used to automatically control the strength of the current in the windings. Such geometry of the heating furnace allowed the temperature of the melts to be maintained within ±1 K, and provided the 70 mm high zone of isothermal heating. The gas space of the cell after loading the salts was sealed and evacuated to a pressure of 0.13 Pa, while the temperature was gradually increased to 573 K, i.e., slightly below the melting point of the lead reference electrode, for 10–12 h. The cell was then filled with purified argon.

### 2.4. Spectroscopy

The surface of the rhenium electrode after anode polarization was investigated by means of the Raman scattering method using a “Leica DMLM” microscope of Raman microscope-spectrometer Renishaw U1000 (Renishaw, New Mills, UK) equipped with an Ar laser with a power density below 40 Wcm^−2^. The scattered radiation was collected at an angle of 180° with respect to the direction of the laser beam.

## 3. Results

**Figure 2:**
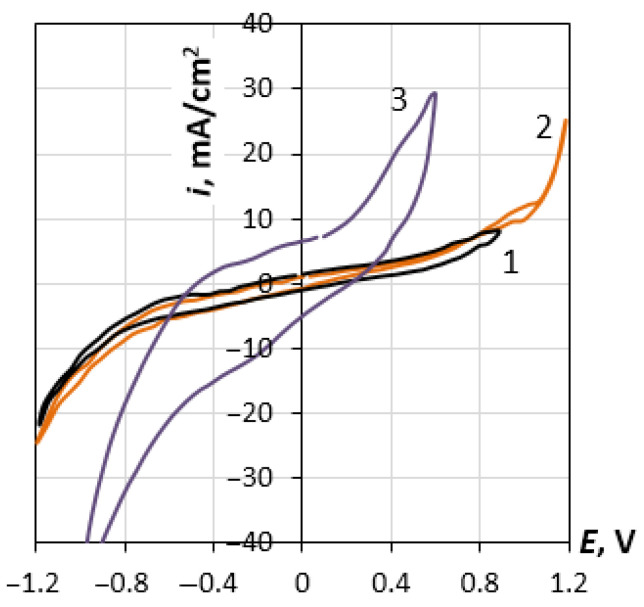
Voltammograms for Re electrode in molten KCl (1093 K). The scanning rate is 5 mVs^−1^ (1), 10 mVs^−1^ (2) and 50 mVs^−1^ (3).

Figure 2 presents voltammograms for the rhenium electrode in the potassium chloride melt at 1093 K and scan rates of 5, 10 and 50 mV/s. The shape of the curve does not change significantly at the rates lower than 10 mV/s. A noticeable hysteresis at high scan rates can be associated with the inhibition of the adsorption–desorption process. Voltammograms for other studied systems have a similar appearance. Based on these studies, we selected the operating range of potentials, where neither active anodic dissolution of rhenium nor cathode deposition of alkali metal was observed.

**Figure 3:**
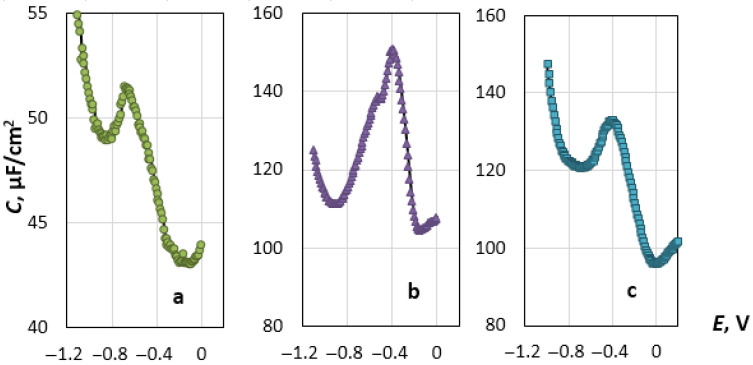
Differential capacitance, *C*, as a function of the potential, *E*, for the Re electrode in (**a**) NaCl, (**b**) KCl and (**c**) CsCl melts at 1093 K.

The primary results are shown in Figure 3 in the form of the dependences of the capacitance, *C*, of the double electric layer (DEL) on the applied potential, *E.* At the potential of the cathode minimum, *E_m_*, apparently, the sign of the charge of the electrode surface changes, as it is predicted by the Gouy-Chapman-Stern (GCS) theory. In its physical sense, *E_m_* is similar to the zero-charge potential (ZCP), determined by the electrocapillary measurements [32]. This statement is based on the experimentally obtained data on the properties of DELs on gold, silver and copper electrodes in salt melts at different temperatures [22,23,33,34]. However, it is necessary to keep in mind the well-known fact that *E_m_* shifts towards negative values relative to ZCP as the frequency of the alternating signal imposed on the base voltage increases [35,36]. Until now, the value of the potential, at which the sign of the charge of the rhenium surface changes to the opposite, has been unknown. The potential is found to shift in the positive direction when the temperature increases, as in the case of ZCP of liquid and solid metals in ionic melts [32,37].

In the vicinity of the *C-E* curve minimum, the capacitance increases as the electrode potential shifts both in the negative direction and (up to a certain point) towards positive values, which is fully consistent with the GCS theory [38]. It is significant that on the anode branch of the *C-E* curve, the capacitance increases as the potential shifts towards positive values, forming a clear “dome” at *E_c_*. At more positive potentials, the capacitance decreases sharply and reaches a minimum value, after which it begins to grow again. The experimental curves resemble those obtained for platinum and gold electrodes in the alkali halide melts [19,20,21,24,25,38]. Such an anomaly was not observed on the silver and copper electrodes [25,26].

## 4. Discussion

The reason for the anomalous behavior of the interfaces in salt melts lies, apparently, in the peculiarities of the salt phase ions on the electrified metal surface. Conventionally, the interface between metal and the condensed salt may be associated with a flat capacitor with

*C = εε_0_/L* (1)

here, *ε* is the dielectric constant of the medium, *ε_0_* is the permittivity of the free space and *L* is the effective length of the double electric layer in the direction perpendicular to the electrode surface, including its dense and sign-oscillating parts [39].

Assuming that the value L covers the entire thickness of the salt part, where the sum of charges compensates completely for the charge of the electrode, it seems reasonable to consider it as the Debye shielding length. For a 1-1 electrolyte charging,

*L = (εε_0_kT/e^2^ρ)^1/2^*, (2)

where *k* is the Boltzmann constant, *T* is the absolute temperature, *e* is the electron charge and *ρ* is the ion packing density. At first approximation, it can be assumed that the increase in capacitance, when the potential is displaced relative to *E_m_*, is associated with the deformation of ions and interparticle bonds in the DEL field [40,41]. For the anode branch of the experimental curves, the change in the layer length:

*∆L = (L_Em_ − L_E_)*, (3)

is estimated, where *L_E_* and *L_Em_* are the effective lengths of DEL at the current potential and at the minimum point of the capacitance curve, respectively. It is assumed that *ε* = 1. It is difficult to say now how this assumption relates to reality, since nothing is known about the value of *ε;* still, less information is available about its dependence on the field strength in DEL. 

**Figure 4:**
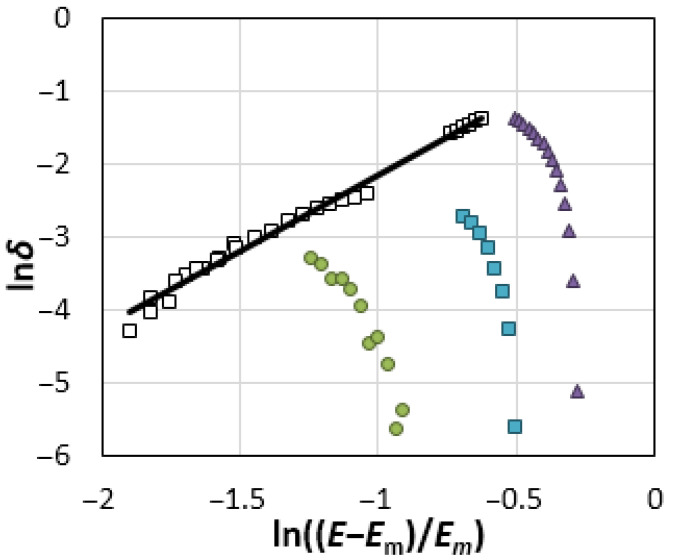
The dependences of the deformation coefficient of DEL *δ = ∆L*/*L*_Em_ on the reduced potential (*E* − *E_m_*)/*E_m_* in the logarithmic form for the Re electrode in NaCl (●), KCl (▲) and CsCl (■) melts (with potentials more positive than the *E_c_*) and in the range of potentials from *E_m_* to *E_c_* (□) at 1093 K.

The calculation results are shown in Figure 4 in the form of the logarithmic dependences of the deformation coefficient of DEL:

*δ = ∆L/L_Em_* (4)

at 1093 K on the reduced potential:

*(E − E_m_)/E_m_*. (5)

As can be seen, there is a potential region where the thickness of DEL decreases as the potential shifts in the positive direction. The observed effect may be caused by an increase in the polarization of ions and chemical bonds as the strength of the electric field between the DEL plates increases.

Since the interionic Coulomb interactions in the studied salts are ordinarily represented by a universal pair potential [42], the entire array of points on the ascending curves is approximated by a single equation:

*δ* = [(*E* − *E_m_*)/*E_m_*]^α^. (6)

It is found that the exponent α is equal to 2.11 (the authenticity of approximation, R^2^, is 0.9905). A similar quadratic dependence is determined for the oscillation frequencies of the sulfate and phosphate anions adsorbed on platinum and gold as a function of the applied potential, due to the deformation of the covalent S–O and P–O bonds in an electric field [43,44]. Figure 4 shows that the compression of DEL reaches the limit values at the potential of the anode maximum, *E_c_*. Starting with this potential, the layer length increases sharply, which is likely to be caused by a decrease in the electric field strength in DEL due to the redistribution of ions in the same oxidation state between DEL plates.

**Figure 5:**
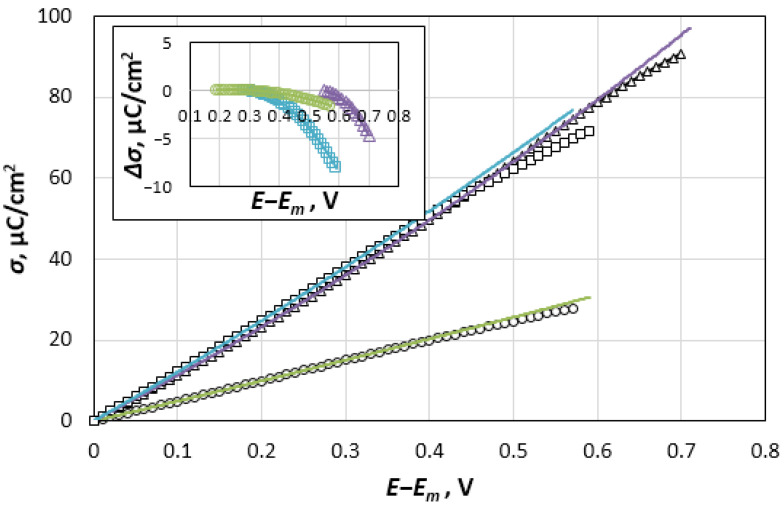
Charge density, *σ*, for the Re electrode as a function of the potential in NaCl (○), KCl (∆) and CsCl (□) melts. Inset: The difference between the real and extrapolated charge density, *∆σ*, at the region of potentials more positive than the *E_c_*.

To substantiate this hypothesis, we present an analysis of the change in the charge density, σ, of the electrode for the anode branch of the curve as a function of the potential. In Figure 5, the charge values detected by integrating the experimental capacitance curves of the studied systems at 1093 K are plotted relative to *E − E_m_*. According to the GCS theory, this dependence should satisfy the exponential equation:

*lnσ* ≈ (*E* − *E_m_*) (7)

in the potential range from *E_m_* to *E_c_* (the potential of the dome top of the capacitance curve). In reality, this equation turned out to be completely inapplicable for describing the experimental curves. With an approximation R^2^ = 1, the experimental σ-E dependences in these potential regions are transmitted by quadratic equations:

Re-NaCl σ = 5.517E^2^ + 48.494E + 0.0091 Re-KCl σ = 40.668E^2^ + 107.69E + 0.0728 Re-CsCl σ = 28.882E^2^ + 118.07E + 0.0569

The extrapolation of these dependences to the region of potentials more positive than *E_c_* showed that the real charge density, σ, is less than the extrapolated value, σ_ex_. The difference between these values, ∆σ, which increases as the potential shifts relative to *E_c_* (Figure 5, inset), indicates a change in the nature of the DEL charging. Considering the systems with potassium and cesium chlorides, the charge density decreases by 5 µC cm^−2^ as the potential shifts by 150 mV in a more positive direction than *E_c_.* In principle, this shift may be caused by the process of rhenium anode dissolution. However, the experimentally observed zero-polarizing currents near *E_c_* (see Figure 2) contradict this hypothesis. To fulfill the local electroneutrality condition, the charge density on the rhenium electrode in the anode region, σ, which is caused by the excess Gibbs energy of cations, zFГ_Re_^z+^, is supposed to be completely shielded by charges of the opposite sign located in the salt part of DEL, which are formed by the excess Gibbs energy of halogen anions, −zFΓ_Cl_^−^. The displacement of the potential in the positive direction leads to an obvious increase both in Г_Re_^z+^ and in Γ_Cl_^−^, as well as to an increase in the interaction energy between the phases. It is the inclusion of this interaction in the Monte Carlo and molecular dynamics calculations [27,28,29] that makes it possible to predict the experimentally observed dome-shaped capacitance curves for positively charged electrodes. However, the approach used to describe the DEL properties in the mentioned papers can hardly predict the formation of complex salts from metal cations and halide anions at the metallic surface [17]. Perhaps this may be explained by the type of the interparticle interaction at the phase contact. Therefore, the pair potential presented in [29] includes the Coulomb ion-electrode attraction and Lennard-Jones repulsion, whereas the interaction model presented in [27] includes the induction of dipoles on the ions of the salt phase, as well as the polarization of metal atoms by molten salt ions. Further development of the theory might require the quantum chemical analysis.

The energy of the capacitor cannot reach infinite values, so that the strengthening of the interaction between Re^n+^ and Cl^−^ ions can generally promote the ions’ transition through the energy barrier across the interface and, thereby, reduce the total energy of the system. It can be assumed that the deficit of the positive charge density shown in Figure 5 is caused by the transfer of a part of the charges, −FA_Cl_^−^, from the salt part of DEL, −zFΓ_Cl_^−^, to the electrode. The charges transferred by the anions cause the appearance of a negative component of the capacitance in the region of potentials that are more positive than *E_c_*. The total charge per unit surface of the electrode should be represented as:

Q = zFГ_Re_^z+^ − FA_Cl_^−^. (8)

The reproducibility of the capacitance curves with altered polarization indicates the reversibility of ion transport in the studied range of potentials.

To identify the form of existence of chlorine ions in the adsorption layer, the chemical nature of rhenium should be taken into account. According to the literature data, rhenium exists mainly in the form of Re (III) and (IV) in chloride melts [45,46,47], with Re (III) being more stable in the NaCl melt, and Re (IV) being more stable in KCl and CsCl. It can be expected that some chemical compounds of rhenium with the chlorine anion may form at the surface of the rhenium electrode.

The principal possibility of the existence of chemical compounds on a positively charged metal surface is proved by direct spectroscopic methods using a gold electrode in halide salt solutions [48,49,50,51]. Considering a positively charged surface of the gold electrode, the following facts are established: (i) vibration modes responsible for the coupling of chlorine ions with gold are detected, (ii) the fixed frequency lines refer to the valence vibrations of the [AuCl_2_]^−^ grouping and (iii) the process of formation and disintegration of groupings is reversible with respect to the potential.

**Figure 6:**
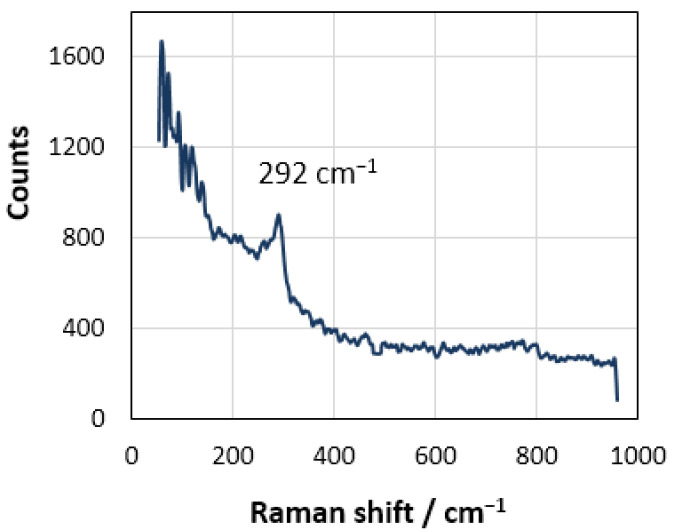
Raman spectra for the Re electrode surface after anodic polarization in the molten potassium chloride (Renishaw Raman microscope-spectrometer U 1000 equipped with a confocal Leica DML microscope, argon laser with a power density below 40 Wcm^−2^).

Unfortunately, the technical problems of setting up such experiments at high temperature have not been overcome yet, mainly due to the lack of suitable construction materials and equipment. Nevertheless, we tried to determine the form of the existence of the chlorine anion on rhenium by the ex situ spectroscopic studies of the electrode after anodic polarization at room temperature. Figure 6 shows the Raman spectrum of the studied electrode surface after anodic polarization in the potassium chloride melt. The Raman-active mode detected at 292 cm^−1^ is explained by the Re–Cl stretching vibration [52]. According to the theory of hybridization of atomic orbitals, the presence of Cl^−^ ligands should promote the mixing of s-, p-, and d-orbitals of rhenium, leading to the formation of four hybrid dsp^2^ orbitals [53]. As a result, it can be expected that [ReCl_4_]^−^ complex anions, having the form of a flat square or [Re_2_Cl_8_]^2−^ clusters [54,55], are formed on the electrode. At a certain anode current density and temperature, these complex anions can acquire the form of RReCl_4_ salts (here, R is an alkali cation), which leads to the passivation of the electrode. Thus, the *E_c_* potential may be considered a critical point of transition from an electrostatic adsorption to a chemical one.

A quantitative theoretical analysis of the mechanism of ion transfer at the electrode/electrolyte interface requires a strong quantum-mechanical calculation. To develop recommendations for optimizing the process of rhenium anode dissolution, it is proposed to study the temperature dependence of the transition from one type of adsorption to another.

## 5. Conclusions

The values of the differential capacitance of the double electric layer (DEL) on a solid rhenium electrode in molten sodium, potassium and cesium chlorides were obtained in a wide range of potentials using the method of impedance spectroscopy. Along with the classical minimum potential, the maximum potential was found in the anode region of the potential dependence of the capacitance. It is assumed that in the vicinity of the minimum capacitance, the behavior of DEL is mainly influenced by the electrostatic polarization of the ions under the action of Coulomb forces. It was shown that this effect is directly proportional to the square of the applied potential for all studied salts. At the critical potential *E_c_*, the maximum electric field strength was reached. Therefore, the transfer of a part of anions from the salt phase to the electrode becomes energetically advantageous. 

The analysis of the dependence of the charge density on the electrode revealed the effect of shielding of potential-induced rhenium cations by salt phase anions.

The product of the interaction between rhenium cations and transferred chlorine anions was recorded by ex situ spectroscopic studies. Of course, a rigorous analysis of the effect of ion polarization on the electric field of DEL at the adsorption stage requires both detailed in situ Raman spectroscopic studies and the corresponding quantum-mechanical calculations, which may be implemented in the future.

## References

[1] Wu W., Chen Z., Wang L. (2015). Oxidation behavior of multilayer iridium coating on niobium substrate. Prot. Met. Phys. Chem. Surf..

[2] Zhu L., Bai S., Zhang H., Ye Y., Gao W. (2014). Long-term high-temperature oxidation of iridium coated rhenium by electrical resistance heating method. Int. J. Refract. Met. Hard Mater..

[3] Huang Y., Bai S., Zhang H., Ye Y. (2015). Oxidation of iridium coating on rhenium coated graphite at elevated temperature in stagnated air. Appl. Surf. Sci..

[4] Huang Y., Bai S., Zhang H., Ye Y., Zhu L. (2016). Oxidation of iridium coatings on rhenium substrates at ultrahigh temperature in stagnant air: Its failure mechanism and life model. Surf. Coat. Technol..

[5] Weiland R., Lupton D.F., Fischer B., Merker J., Scheckenbach C., Witte J. (2006). High-temperature mechanical properties of the platinum group metals. Platin. Met. Rev..

[6] Toenshoff D., Lanam R., Ragaini J., Shchetkovskiy A., Smirnov A. Iridium coated rhenium rocket chambers produced by electroforming. Proceedings of the 36th AIAA/ASME/SAE/ASEE Joint Propulsion Conference and Exhibit.

[7] Zhu L., Bai S., Zhang H., Ye Y., Gao W. (2013). Rhenium used as an interlayer between carbon–carbon composites and iridium coating: Adhesion and wettability. Surf. Coat. Technol..

[8] Kuznetsov S.A. (2009). Electrochemistry of refractory metals in molten salts: Application for the creation of new and functional materials. Pure Appl. Chem..

[9] Isakov A.V., Apisarov A.P., Nikitina A.O. (2017). Electrowinning and annealing of Ir—Re—Ir material. Tsvetnye Met. (Nonferrous Met.).

[10] Baraboshkin A.N. (1976). Electrocrystallization of Metals from Molten Salts.

[11] Timofeyev N.I., Baraboshkin V.E., Saltykova N.A. Production of iridium crucibles by electrolysis of molten salts. Proceedings of the International Symposium, 2000 TMS Annual Meeting.

[12] Huang Y., Bai S., Zhang H., Ye Y. (2015). Growth mechanism and mechanical property of laminar iridium coating by electrodeposition. Int. J. Refract. Met. Hard Mater..

[13] Saltykova N.A., Portnyagin O.V. (2001). Electrodeposition of iridium–ruthenium alloys from chloride melts: The structure of the deposit. Russ. J. Electrochem..

[14] Qian J., Zhao T. (2012). Electrodeposition of Ir on platinum in NaCl-KCl molten salt. Trans. Nonferrous Met. Soc. China.

[15] Huang Y., Bai S., Zhang H., Ye Y., Zhu L. (2017). Electrocrystallization of iridium on the graphite, rhenium and iridium electrode from the NaCl-KCl-CsCl-IrCl3 molten salts. J. Electroanal. Chem..

[16] Chernyshev A.A., Apisarov A.P., Isakov A.V., Zaikov Y.P., Malkov V.B., Laptev M.V. (2017). Rhenium electrowinning in the KF-KBF4-B2O3-KReO4 Melt. J. Electrochem. Soc..

[17] Saltykova N.A., Pecherskaya L.S., Baraboshkin A.N., Kotovsky S.N., Kosikhin L.T. (1986). Salt passivation during anodic dissolution in iridium chloride melts. Elektrokhimiya.

[18] Graves A.D., Inman D. (1965). Adsorption and the differential capacitance of the electrical double-layer at platinum/halide metal interfaces. Nature.

[19] Laitinen H.A., Roe D.K. (1960). Double layer capacity of platinum and bismuth electrodes in molten lithium chloride-potassium chloride. Collect. Czech. Chem. Commun..

[20] Hills G.J., Johnson K.E. (1961). Impedance Phenomena in molten salts. J. Electrochem. Soc..

[21] Bukun N.G., Alekseeva R.A. (1975). Capacitance of the double electrical layer of gold in molten chorides. Russ. J. Electrochem..

[22] Dokashenko S.I., Kirillova E.V., Stepanov V.P. (2004). Impedance of a polycrystalline gold electrode in molten KCl. Rasplavy (Melts).

[23] Kirillova E.V., Dokashenko S.I., Stepanov V.P. (2011). Capacitance of a polycrystalline silver electrode in Na, K, and Cs bromide melts. Russ. Metall. (Metally).

[24] Kirillova E.V., Dokashenko S.I., Stepanov V.P. (2008). Capacitance of a polycrystalline gold electrode in molten alkali bromides. Rasplavy (Melts).

[25] Kirillova E.V., Stepanov V.P. (2016). Capacitance of the double electrical layer on the copper-group metals in molten alkali metal halides. Russ. Metall. (Metally).

[26] Kirillova E.V., Dokashenko S.I., Stepanov V.P. (2007). Capacitance of a polycrystalline silver electrode in Na, K, and Cs chloride melts. Rasplavy (Melts).

[27] Pounds M., Tazi S., Salanne M., Madden P.A. (2009). Ion adsorption at a metallic electrode: An ab initio based simulation study. J. Phys. Condens. Matter..

[28] Kłos J., Lamperski S. (2019). Electrical double layer in molten salts with account of soft repulsions. J. Chem. Phys..

[29] Kłos J., Lamperski S. (2020). Electrical double layer in molten salts taking into account Lennard-Jones potential. Electrochim. Acta.

[30] Warren R.W. (1965). Procedures and apparatus for zone purification of the alkali halides. Rev. Sci. Instrum..

[31] Stepanov V.P., Minchenko V.I. (2013). Ultrasonic velocity for an equimolar mixture of molten AgI and NaCl in the biphasic region. J. Chem. Thermodyn..

[32] Smirnov M.V., Stepanov V.P. (1982). Density and surface tension of molten alkali halides and their binary mxitures. Electrochim. Acta.

[33] Stepanov V.P., Sitnikov L.V. (2014). Surface properties of elastically deformed copper electrode in alkali metal chloride melts. Russ. Metall. (Metally).

[34] Pastukhov Y.G., Stepanov V.P. (1989). Doklady Akademii Nauk SSSR.

[35] Ryabchuk V.N., Stepanov V.P., Belyaev V.S., Filyaev A.T. (2001). Differential surface stress of single-crystal gold(111) in a potassium chloride melt. Russ. J. Electrochem..

[36] Stepanov V.P., Dokashenko S.I., Kirillova E.V. (2012). Frequency dependence of potentials of minimum capacitance for electrodes of copper subgroup metals in alkali halide melts. Russ. J. Electrochem..

[37] Stepanov V.P. (2004). Potential of zero charge in molten alkali and alkaline-earth halides. Rasplavy (Melts).

[38] Tazi S., Salanne M., Simon C., Turq P., Pounds M., Madden P.A. (2010). Potential-induced ordering transition of the adsorbed layer at the ionic liquid/electrified metal interface. J. Phys. Chem. B.

[39] Graves A.D., Inman D. (1970). The electrical double layer in molten salts: Part 2. The double-layer capacitance. J. Electroanal. Chem. Interfacial Electrochem..

[40] Stark J., Wendt G. (1914). Beobachtungen über den Effekt des elektrischen Feldes auf Spektrallinien. II. Längseffekt. Ann. Phys..

[41] Stark J., Kirschbaum H. (1914). Beobachtungen über den Effekt des elektrischen Feldes auf Spektrallinien. III. Abhängigkeit von der Feldstärke. Ann. Phys..

[42] Tosi M.P., Fumi F.G. (1964). Ionic sizes and born repulsive parameters in the NaCl-type alkali halides. J. Phys. Chem. Solids.

[43] Nart F.C., Iwasita T. (1992). Second order Stark effect of adsorbed sulfate ions on polycrystalline platinum. Electrochim. Acta.

[44] Weber M., Nar F.C. (1996). On the adsorption of ionic phosphate species on Au(111)—An in situ FTIR study. Electrochim. Acta.

[45] Stepanov A.D., Vetyukov M.M., Shkolnikov S.N. Corrosion and anodic behavior of rhenium in chloride melts. Proceedings of the V Ural Conference on High-Temperature Physical Chemistry and Electrochemistry, UrO AN SSSR.

[46] Kuznetsov S.A., Kalinnikov V.T. (2011). Basicity of molten salts and stabilization of higher oxidation states of d and f elements. Dokl. Akad. Nauk.

[47] Kuznetsov S.A. (1994). Electroreduction of rhenium complexes in halide and oxyhalide melts. Elektrokhimiya (Electrochemistry).

[48] Loo B.H. (1982). In situ identification of halide complexes on gold electrode by surface-enhanced Raman spectroscopy. J. Phys. Chem..

[49] Gao P., Weaver M.J. (1986). Metal-adsorbate vibrational frequencies as a probe of surface bonding: Halides and pseudohalides at gold electrodes. J. Phys. Chem..

[50] Melendres C.A., Hahn F. (1999). ‘In-situ’ observation of halide ion adsorption on a gold electrode using synchrotron far infrared spectroscopy. J. Electroanal. Chem..

[51] Sitnikov L.V., Zakir’yanova I.D., Kirillova E.V. (2016). Investigation of chloride interaction with electrically polarized gold surface by surface-inhanced Raman spectroscopy. Rasplavy (Melts).

[52] Shimanouchi T. (1977). Tables of molecular vibrational frequencies. Consolidated volume II. J. Phys. Chem. Ref. Data.

[53] Davtyan O.K. (1962). Quantum Chemistry.

[54] Borisova L.V., Ermakov A.N. (1974). Analytical Chemistry of Rhenium.

[55] Huheey J. (1983). Inorganic Chemistry: Princiles of Structure and Reactivity.

